# *SAXSDOG*: open software for real-time azimuthal integration of 2D scattering images

**DOI:** 10.1107/S1600576722003685

**Published:** 2022-05-28

**Authors:** Max Burian, Christian Meisenbichler, Denys Naumenko, Heinz Amenitsch

**Affiliations:** a Institute of Inorganic Chemistry, Graz University of Technology, Stremayergasse 9/IV, Graz, 8010, Austria

**Keywords:** computer programs, small-angle X-ray scattering, SAXS, azimuthal integration, *SAXSDOG*

## Abstract

*In situ* small- and wide-angle scattering experiments at synchrotrons often result in massive quantities of data within just seconds. Especially during such beamtimes, processing of the acquired data online, without appreciable delay, is key to obtaining feedback on the failure or success of the experiment. This had led to the development of *SAXSDOG*, a Python-based environment for real-time azimuthal integration of large-area scattering images. The software is primarily designed for dedicated data pipelines: once a scattering image is transferred from the detector onto the storage unit, it is automatically integrated and pre-evaluated using integral parameters within milliseconds. The control and configuration of the underlying server-based processes is achieved via a graphical user interface, SAXSLEASH, which visualizes the resulting 1D data together with integral classifiers in real time. *SAXSDOG* further includes a portable ‘take-home’ version for users that runs on standalone computers, enabling its use in laboratories or at the preferred workspace.

## Abstract 2

The open-source software *SAXSDOG* performs a real-time azimuthal integration of 2D scattering images reaching peak integration performance at current hardware limits.

## 1. Introduction

Synchrotron radiation sources provide the high flux needed for *in situ* scattering experiments with milli- and microsecond time resolution (Fadenberger *et al.*, 2010 ▸; Golks *et al.*, 2011 ▸; Narayanan *et al.*, 2014 ▸). These experiments are fundamental to study physical, chemical and biological mechanisms occurring at the molecular nanometre level. In particular, small-angle X-ray scattering (SAXS) is among the few techniques offering structural insight into these phenomena (Li *et al.*, 2016 ▸; Kikhney & Svergun, 2015 ▸). For standard [non-stroboscopic (Levantino *et al.*, 2015 ▸) and/or continuous-flow (Marmiroli *et al.*, 2009 ▸)] experiments, the best achievable time resolution is limited by the readout time of the X-ray detector, which is of the order of milliseconds (DeCaro *et al.*, 2013 ▸; Kocsis *et al.*, 2006 ▸; Johnson *et al.*, 2014 ▸; Kraft *et al.*, 2009 ▸; Delogu *et al.*, 2016 ▸; Ponchut *et al.*, 2011 ▸). However, even frame rates of 100–1000 Hz produce massive quantities of data, considering that 2D detectors often consist of more than 1 megapixel (Tuukkanen *et al.*, 2017 ▸). This raises the demands on the hard- and software used in the data-processing pipeline, which are the backbone of stable and efficient beamline operation.

In SAXS and powder diffraction, the experimentally recorded 2D scattering images have to be transformed into 1D scattering patterns by means of azimuthal integration (Hammersley *et al.*, 2007 ▸; Boesecke, 2007 ▸; Rodriguez-Navarro, 2006 ▸; Sztucki & Narayanan, 2007 ▸; Ren *et al.*, 2017 ▸). When done manually, this operation can be time consuming and hence cause unused dead-time of user-dedicated ring operation. Several beamlines and synchrotrons have developed custom solutions to accelerate and/or automate this integration process (Benecke *et al.*, 2014 ▸; Kieffer & Karkoulis, 2013 ▸; Ilavsky, 2012 ▸; Brennich *et al.*, 2016 ▸; Hopkins *et al.*, 2017 ▸; Franke *et al.*, 2012 ▸), resulting in impressive computational performance close to current hardware limits (Ashiotis *et al.*, 2015 ▸). As, however, each beamline is unique in regard of (*a*) its user base, (*b*) the performed experiments and hence (*c*) the demands on data processing, we aimed to develop a framework that (i) operates automatically on the beamline’s data backbone, (ii) is configurable via a user interface and (iii) processes data in (close to) real time. The side benefit of this vision is that the reactions/phenomena measured during the experiment can be monitored online via data classifiers (Glatter & Kratky, 1982 ▸; Feigin *et al.*, 1987 ▸), providing rapid feedback on the experimental conditions without further, often time-consuming, data evaluation.

In this work, we present *SAXSDOG*, an open-source Python-based program designed for fast online azimuthal integration and pre-evaluation of 2D scattering images, which is currently in operation at the Austrian SAXS beamline at Elettra. *SAXSDOG* offers two modes of operation: (1) a ‘local-server mode’ that can be run on standalone computers and (2) a ‘remote-server mode’ incorporated in the data pipeline of our endstation, under which it reaches its full potential. We explain the common subroutines of both operation modes and focus on the details for the implementation of *SAXSDOG* in a performance server network as found at common beamlines. We further show how the server process is controlled and configured via the Qt-based graphical user interface (GUI) SAXSLEASH, which is also used to visualize the integrated data as well as the corresponding integral parameters. These merits will be emphasized by means of a given example, which will also demonstrate how *SAXSDOG* can help to get a first glimpse of the studied effects without extensive data evaluation.

## 2. Specifications

The *SAXSDOG* package is written in Python (v3.5) and has been developed in the Anaconda framework. During the development, special care has been taken to make the software, and the included dependencies, cross-platform compatible. A detailed list of all package versions for stable operation can be found in the user manual that is distributed with the source code. The most fundamental packages are (i) Qt4 (providing the GUI as well as the signalling protocol between threaded processes), (ii) JSON schema (providing the data standard on server and client), (iii) PyZMQ (providing the network-communication standard) and (iv) Pillow (providing the Python image-processing library). The maintained version of *SAXSDOG* can be downloaded from GitHub at https://github.com/maxburian/SAXS_py3 and includes a user manual (local web site) with step-by-step installation instructions and more detailed information on the source code. We explicitly encourage users to participate in further code development via the GIT platform. The software can be used and is released free of charge under the GNU General Public License.

## 3. Principles – the *SAXSDOG* network

**Figure 1:**
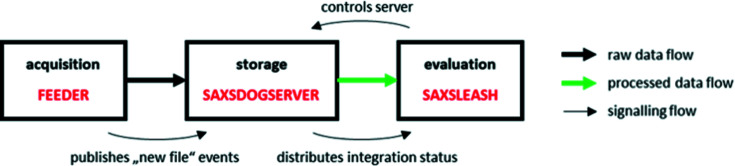
Data and signalling flow of the *SAXSDOG* software package.

The functionality of the software package is based on the server–client principle summarized in the *SAXSDOG* network shown in Fig. 1 ▸. In the following, the data and control flow of the pipeline are explained.

Once an image is acquired by the detector (here, a Pilatus3 1M; Dectris, Switzerland), it is automatically transferred from the temporary detector storage (here, the Pilatus Processing Unit, PPU) to the beamline’s (long-term) data-storage server (see bold black arrow in Fig. 1 ▸). This file-transfer script (here called FEEDER) includes a command that publishes a ‘new file’ event over the network via ZMQ message. On the data-storage server, the SAXSDOGSERVER waits for such a ‘new file’ event from the FEEDER, as it signals which image to integrate next. Once the integration is completed, the processed data together with additional data classifiers (see bold green arrow in Fig. 1 ▸) are stored and distributed in real time to the GUI on the client/user PC: the SAXSLEASH.

The core module of this processing pipeline is the SAXSDOGSERVER, a daemon process running on the beamline’s data-storage server. The SAXSDOGSERVER is controlled by the SAXSLEASH over an HTTP/REST interface, which sets the integration parameters and (de-)activates the processing queue. The underlying network architecture is defined by the $home/.saxsdognetwork file (which can be called via the saxsnetconf command), specifying the IP addresses of the FEEDER and SAXSDOGSEVER as well as an authentication secret (which de- and encrypts the sent and received message, respectively). As all three server instances communicate via open and by default unrestricted data protocols (HTTP/REST and ZMQ) no prior mounting of the servers is necessary. The precise functionality of all three modules is explained in the following subsections.

### 3.1. FEEDER

**Figure 2:**
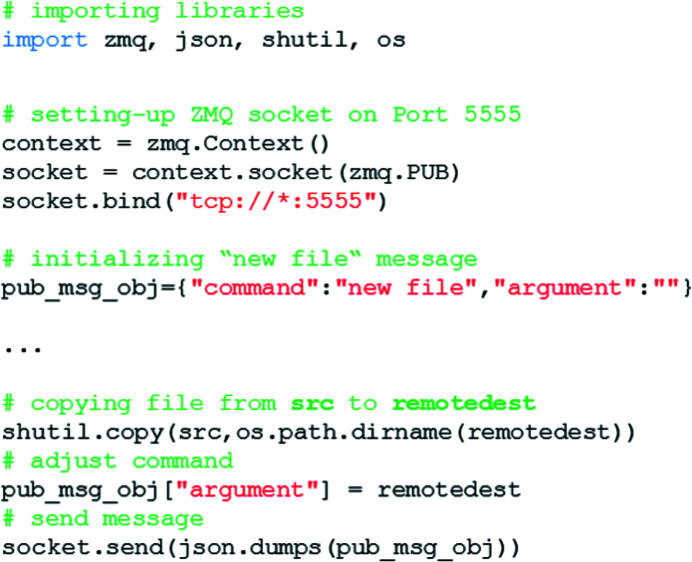
Example of the core code necessary to run the FEEDER service.

The SAXSDOGSEVER subscribes to the FEEDER: a script distributing ‘new file’ events over the network. Such an event consists of a ‘command’ (‘new file’) and an ‘argument’ (path\to\image\file\on\storage\server), packaged in a Python dictionary. Once the image has successfully been copied from the temporary to the data-storage server, the ‘new file’ command is rendered [(i) adding the remote file path and (ii) encoding it to JSON] and sent via ZMQ (we use port 5555). In our implementation of *SAXSDOG* at the Austrian SAXS beamline, we have customized the GRIMSEL service provided by Dectris (Switzerland), which is responsible for transferring acquired images from the temporary storage (PPU) to the beamline’s data-storage server. The ‘new file’ command is only sent once the image has been written successfully, as otherwise the SAXSDOGSERVER accesses a non-existing or non-complete file on the storage server. An example of such a *FEEDER* script is shown in Fig. 2 ▸.

### 3.2. SAXSDOGSERVER

**Figure 3:**
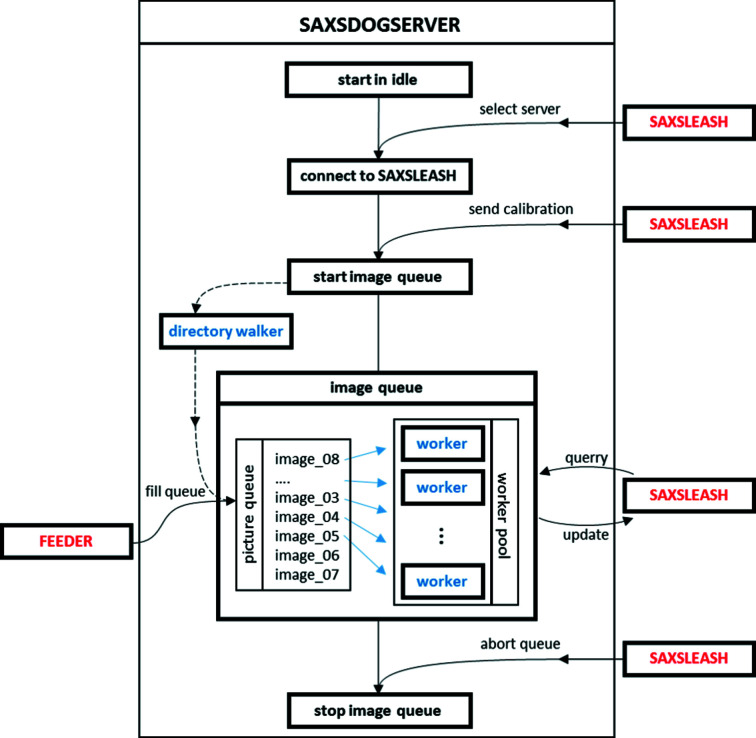
Internal workflow of the SAXSDOGSERVER. Segments in red refer to external processes (FEEDER and SAXSLEASH); segments in blue refer to internal processes (controlled by SAXSDOGSERVER). Lines with arrows indicate internal and external communication flow – dashed lines are optional procedures.

The SAXSDOGSERVER is the core module of the *SAXSDOG* software package as it performs all computational data-processing steps. A graphical overview of the working principle is given in Fig. 3 ▸, which is explained in detail in the following subsection.

The SAXSDOGSERVER is designed to run as a background service on the processing node of the data-storage server. When started, the process is idle, waiting for a SAXSLEASH to connect. The connection is only possible if the authentication secret de- and encrypting the network communication is identical on both machines. If the connection is established, the SAXSDOGSERVER waits for the integration calibration, which defines the geometry, integration mask, directory *etc.* (see Section 4 for details). Once SAXSLEASH sends the ‘new’ queue command, the actual integration process, the ‘image queue’, is initialized.

The image queue is a threaded process that synchronizes two main modules: (*a*) the picture queue and (*b*) the worker pool (see Fig. 3 ▸). In regard of (*a*), the picture queue collects all filenames of the images that need to be processed in a central register. The picture queue is filled by either (i) the FEEDER (for freshly acquired images) or (ii) a directory walker (recursively identifying all existing images in the chosen folder path). In regard of (*b*), the worker pool consists of a user-defined number of parallel ‘workers’ that perform the actual image processing. Each worker takes one image after the other from the picture queue, integrates it and stores the processed data (*.chi file). In addition to the image integration, the workers also calculate image classifiers (*e.g.* integral parameters; Feigin *et al.*, 1987 ▸; Glatter & Kratky, 1982 ▸) of the scattering data and store them in the SAXSDOGSERVER temporary memory. These data may be queried from the SAXSLEASH at any moment, such that the integration progress can be monitored and preliminary data evaluation is possible online, so simultaneously to image acquisition. The image queue stays active until the ‘abort’ command is sent from SAXSLEASH or until the SAXSDOGSERVER is terminated.

### 3.3. SAXSLEASH

**Figure 4:**
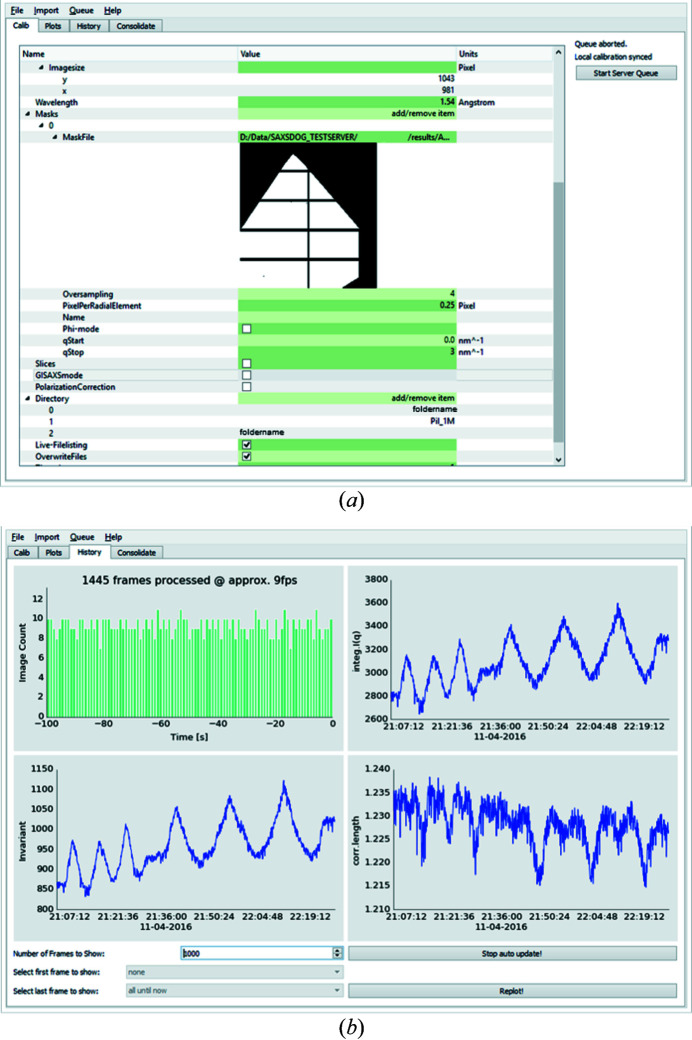
Screenshot of the SAXSLEASH GUI. (*a*) The calibration tab allows the user to display and alter all required and optional integration parameters. (*b*) The history tab shows the current progress of the integration on the SAXSDOGSERVER [progress histogram (top left)] and visualizes the calculated image classifiers [integral intensity (top right), invariant (bottom left) and correlation length (bottom right)].

The SAXSLEASH is a graphical user interface (GUI) that fulfils three main purposes: (i) setting up the integration calibration, (ii) controlling the SAXSDOGSERVER and (iii) monitoring the integration status. In regard of (i), a calibration editor allows the display and alteration of all required and optional integration parameters [see Fig. 4 ▸(*a*)] and selection and display of image-masking files (*.msk output from *FIT2D*). To simplify the input, we included converter tools such that the calibrated geometry values from *FIT2D* (Hammersley, 2016 ▸; Hammersley *et al.*, 2007 ▸) or *NIKA2D* (Ilavsky, 2012 ▸) can be converted into the *SAXSDOG* format (see Section 4 for details). In regard of (ii), the SAXSDOGSERVER may be controlled by (*a*) sending a new integration calibration, which starts a new image queue, (*b*) forcing a reintegration of all existing image files (starts directory walker as shown in Fig. 3 ▸), and (*c*) aborting and clearing the current image queue. In regard of (iii), the current status of the integration on the SAXSDOGSERVER can be monitored in the ‘Plots’ and ‘History’ tabs [see Fig. 4 ▸(*b*) for history output on example data]. In the histogram shown in the top left, the integration progress and the integration speed are displayed. The other three panels show the image classifiers (here integral parameters) over the time the image was acquired (taken from the image header), allowing a glimpse of, for example, reaction dynamics without further data evaluation. Using the selection tool at the bottom of the window, the user can display the classifier values of a single data set only, without having to reintegrate the entire image queue.

### 3.4. Local-server mode

Instead of running all three *SAXSDOG* modules, *i.e.* FEEDER, SAXSDOGSERVER and SAXSLEASH, on separate machines, we implemented a ‘local-server mode’ that automatically emulates a working network on a single machine. The local server can be selected as a starting option when SAXSLEASH is run. When this mode is selected, the user needs to specify a ‘working directory’, which acts as the root directory of the hidden SAXSDOGSERVER process. The main difference from the dedicated implementation is that the local server cannot be run together with the FEEDER (see Fig. 3 ▸), such that only existing image files can be integrated (only the directory walker fills the image queue – see Fig. 3 ▸). However, this program option is ideal for beamline users to take home or for laboratory machines, where ease of use has higher priority than integration speed (see Section 5 for performance metrics).

## 4. The detector calibration

The ‘calibration’ defines the experimental parameters required for adequate image processing on the SAXSDOGSERVER. It includes all necessary metadata information (*e.g.* X-ray energy, scattering geometry, detector parameters *etc*.) for azimuthal averaging, scattering-angle to scattering-vector conversion, and image masking and processing options. A list of all required (mandatory) parameters can be found in Table 1 ▸ (refer to the program manual for a description of optional parameters). The calibration is stored internally as a dictionary-type variable and is saved (as a file) or communicated (sent via ZMQ from SAXSLEASH to SAXSDOGSERVER) according to the JSON structure. The corresponding file for a specific experiment must hence be written in JSON code, but the SAXSLEASH provides a GUI for creating and editing such files without manual coding.

In order to help users better understand the functionality of the integration parameters, the following subsections will explain the underlying detector geometry, how the azimuthal integration is implemented, and how horizontal and vertical slices can be used to evaluate grazing-incidence SAXS (GISAXS) experiments.

### 4.1. The geometry

**Figure 5:**
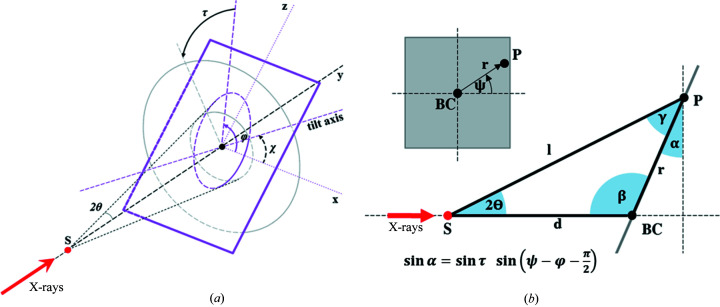
(*a*) Sketch of the geometry convention used in *SAXSDOG*. Here, the X-ray primary beam (bold red arrow) impinges on the sample (red dot) along the *y* axis. The detector (violet plane) is tilted away from the primary-beam trajectory (the normal incidence plane is shown in grey). The detector tilt can hence be defined in terms of two unique angles according to *FIT2D* definitions: (i) the tilt rotation φ [violet arc, φ = χ + π/2 with χ as the angle between the tilt axis and detector horizontal axis as used in *GSAS-II* (Toby & Dreele, 2013 ▸)] and (ii) the tilting angle τ (black arc). The positive directions of angles are indicated by arrows. The distortion effect of these two tilting angles can, however, be summarized such that the 3D geometry can be reduced to a 2D geometric problem for each detector pixel in terms of α as shown in (*b*). Here, detector pixels are expressed in distance *r* from the beam center (BC) and angle from the horizontal axis ψ (see inset).

For ease of operation, SAXSDOG uses the same detector geometry convention as *FIT2D* (Hammersley *et al.*, 2007 ▸). While parameters such as sample-to-detector distance, beam center, image size and pixel size are self-explanatory, special care has to be taken when working with tilted detectors. Here, we consider the tilted detector plane with respect to the normal incidence plane. This detector tilt is defined by two angles: (i) the tilt rotation φ (‘rotating angle of tilting plane’ in *FIT2D*) and (ii) the tilting angle τ (‘angle of detector tilt in plane’ in *FIT2D*). A sketch of this geometry is shown in Fig. 5 ▸(*a*).

The 3D detector geometry is reduced to a 2D geometric problem by assigning two values to each detector pixel: (i) the distance *r* between the beam center (BC) and pixel (P), and (ii) the azimuthal angle ψ between **r** and the horizontal detector axis [see inset in Fig. 5 ▸(*b*)]. In this case, the detector tilt rotation φ, the detector tilting angle τ and the azimuthal angle ψ can be summarized in terms of a single distortion angle α [see Fig. 5 ▸(*b*)], which can be written as

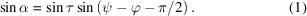

If the distortion angle α, sample-to-detector distance *d* and pixel position *r* are known, the scattered light path *l* can be obtained via

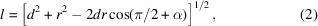

which is then used to determine the scattering angle 2θ and the scattering vector magnitude *q* according to

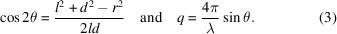




### 4.2. Azimuthal integration

The azimuthal integration in *SAXSDOG* is implemented in a matrix–vector multiplication scheme (similar to *pyFai*; Kieffer & Wright, 2013 ▸) which reassigns pixels from the 2D image to *q* bins in one-dimensional vector format. The computational implementation goes as follows. Every image *p* [size: 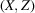 ] is reshaped into a 1D vector of pixels 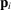 [size: 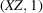 ] such that the scattering intensity of each pixel is addressable by a single index *i*. In this scheme, the integration in a certain radial interval of the image can hence be seen as the weighted sum of all pixels: pixels within the radial element are weighted by 1 and pixels outside are weighted by 0. For a single radial element *j*, the mean intensity 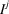 can be calculated by vector–vector multiplication (dot product) of the weighting vector 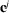 [size: 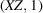 ] with the image vector 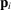 via

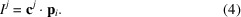

As *SAXSDOG* intends to obtain all 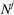 radial elements at once, this multiplication can be rewritten in the final matrix–vector dot-product form

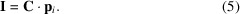


**C** [size: 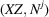 ] is the calibration-dependent weighting matrix and **I** [size: 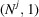 ] is the azimuthally averaged scattering-intensity vector, where the *j*th entry corresponds to the *j*th radial element (so 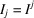 ). Masked pixels (as defined by calibration element masks – see Table 1 ▸) are set to ‘NaN’ in the weighting matrix **C**, such that they are ignored in the matrix–vector product of equation (5). As most entries of the weighting matrix **C** are in fact zeros, we implement the computation in a sparse-matrix representation, which significantly speeds up computation and reduces the memory overhead.

The azimuthal integration also includes the calculation of the correct error band, here dominated by the random nature of scattering events that are best described by a Poisson distribution (Sedlak *et al.*, 2017 ▸). This Poisson error can be calculated for each radial element from (i) the mean intensity (obtained by azimuthal integration above) and (ii) the integration area of the radial slice. The integration area 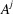 is obtained from the number of counted pixels within the *j*th radial element such that an area-vector **A** [size: 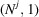 ] can be obtained in analogy to the above by the matrix–vector dot product of the weighting matrix **C** with a unit vector **1** [size: 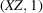 ] by

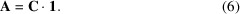

The error vector *E* [size: 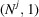 ] corresponding to the azimuthally averaged scattering intensity **I** is then calculated for each image by elementwise multiplication (denoted as 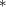 ) according to

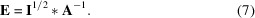

**Figure 6:**
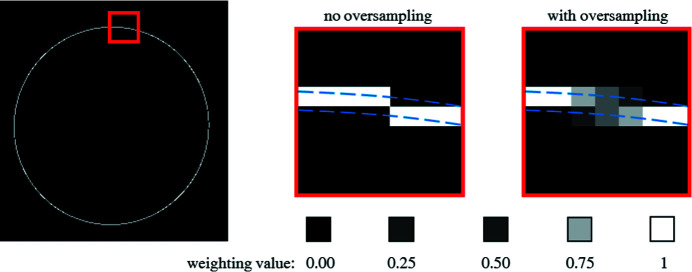
Example of the problematic nature of discretization effects in azimuthal averaging of 2D detector images. The left image shows all pixels in an example image that contribute to the azimuthal integration of a single radial element, where the red box highlights the region of interest. Here, integration within the geometrically defined radial segment (dashed blue lines) causes artefacts as in some cases it is not clear which pixels to count in- or outside of the integration area. Oversampling mitigates this issue, as non-binary weighting values allow that a single pixel can be considered for two radial segments but with corresponding weight.

The matrix–vector multiplication has been extended with an oversampling/anti-aliasing scheme that mitigates discretization effects in 2D image integration. An example of this oversampling scheme for a single radial element (here assuming no detector tilt) is shown in Fig. 6 ▸. Considering a radial segment with a width of a single pixel (see blue lines in Fig. 6 ▸), one encounters two problems in azimuthal integration: (i) a single pixel might lie on the border of two segments and (ii) a radial segment might run through two pixels. By only choosing the nearest pixel for integration (see ‘no oversampling’ in Fig. 6 ▸), one may induce artefacts in the resulting curve, especially when only a few pixels contribute (*e.g.* close to the beam center in the small-angle scattering region). Here, we use an algorithm similar to anti-aliasing in computer graphics (Rosow & Burns, 2004 ▸), where we divide a much larger reference image (multiples of detector size) into the radial intervals and downsample the segments to the original image size. This yields a non-binary weighting matrix **C** for integration [see equation (5)] that consists of intermediate weighting values between 0 and 1 such that the intensity is conserved after averaging.

### 4.3. GISAXS slices

**Figure 7:**
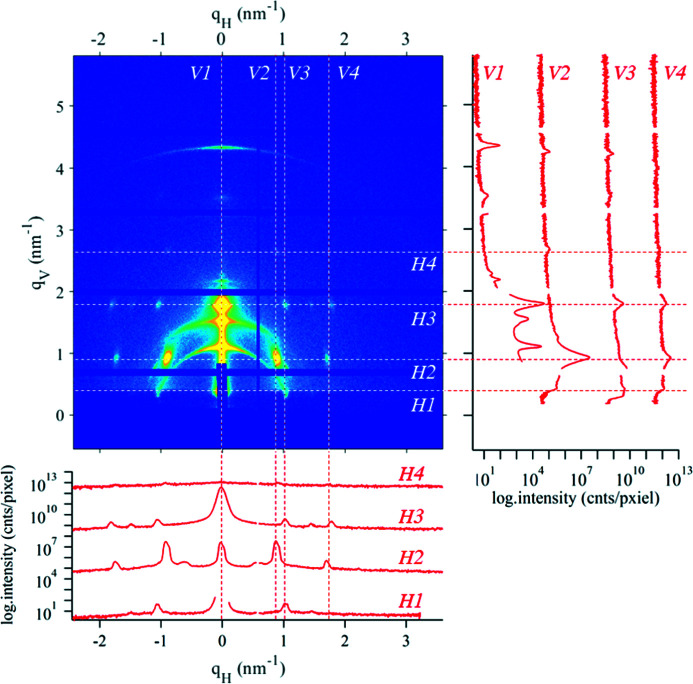
Example of how multiple slices may be placed in detector images, here in a GISAXS pattern of nanostructured lipids on a mesoporous SiO_2_ matrix. Horizontal slices (direction: x and plane: InPlane) and vertical slices (direction: y and plane: Vertical) are calculated within the detector system **q**_H_ and **q**_V_ and do not include the Ewald-sphere distortion correction. Dashed lines mark the slice-position, where each slice has a margin of 7 pixels (corresponding to a thickness of 2 × 7 + 1 = 15 pixels).

For the rapid evaluation of experimental scattering data from grazing-incidence SAXS (GISAXS) experiments, we included the option in *SAXSDOG* to calculate horizontal and vertical cuts of the detector image, termed ‘slices’. Such slices are defined as an array of slice objects in the calibration – an overview of the necessary parameters can be found in Table 2 ▸. An example showing multiple slices within a single image is shown in Fig. 7 ▸.

As known, grazing-incidence experiments do not probe directly along the **q** *_Z_* direction in reciprocal space (Müller-Buschbaum, 2009 ▸; Hexemer & Müller-Buschbaum, 2015 ▸). Moreover, in not perfectly planar samples, the incidence angle (a critical parameter in performing the correct *q*-space conversion) is calculated from the specular peak position after measurements have been made and not at the moment when the integration calibration is defined. A single set of integration parameters that convert each image into ‘true’ reciprocal set parameters would hence induce a wrong *q* scaling in the data-treatment pipeline and would make the sliced data prone to misinterpretation. *SAXSDOG* therefore treats slices only in the detector coordinate system, so without Ewald-sphere distortion correction (and neglecting the incidence angle of the X-ray beam), such that it calculates the mean scattering intensity along the vertical- and horizontal-scattering components on the detector **q**_V_ and **q**_H_, respectively. Note that this assumption is only valid for small scattering angles or when samples are disordered in plane and only partially aligned out of plane. For more ordered samples, we at this point refer to specialized programs for a correct and precise treatment of the *q*-space distortion for single images (Pandolfi *et al.*, 2018 ▸; Jiang, 2015 ▸; Lilliu & Dane, 2015 ▸). However, for the evaluation of GISAXS data during beamtimes, which is of peculiar interest for *in situ* and *operando* experiments, the availability of sliced image data through an automated data pipeline is of high value by drastically facilitating the optimization of measurement conditions.

## 5. Performance

We benchmark the performance of *SAXDOG* in terms of ‘frames per second’ (f.p.s.) by measuring the total time required to integrate a set of images acquired with a Pilatus 1M (Dectris, Switzerland – 1 megapixel, uncompressed TIFF file format, 4 MB per file). The image acquisition rate was set at 200 Hz. The integration calibration included a dead-pixel mask only, such that the entire scattering image is integrated and therefore the size of the sparse weighting matrix (see Section 4.2) is kept at the maximum. The number of workers in the image queue performing the image processing (see Fig. 3 ▸) is hence the main variable of this benchmarking test. For each server configuration, three separate measurements (to estimate variability) consisting of 5000 detector images each were made. Performance tests have been run on (i) the beamline server (dual socket, Intel Xeon E5-2650v4, 12-core @ 2.2 GHz, 24 × 10 TB HDD Seagate ST10000NM0016) and (ii) a workstation laptop (Intel i7-4800MQ, four-core @ 2.7 GHz, 1 × 500 GB HDD Toshiba MQ01ACF050).

**Figure 8:**
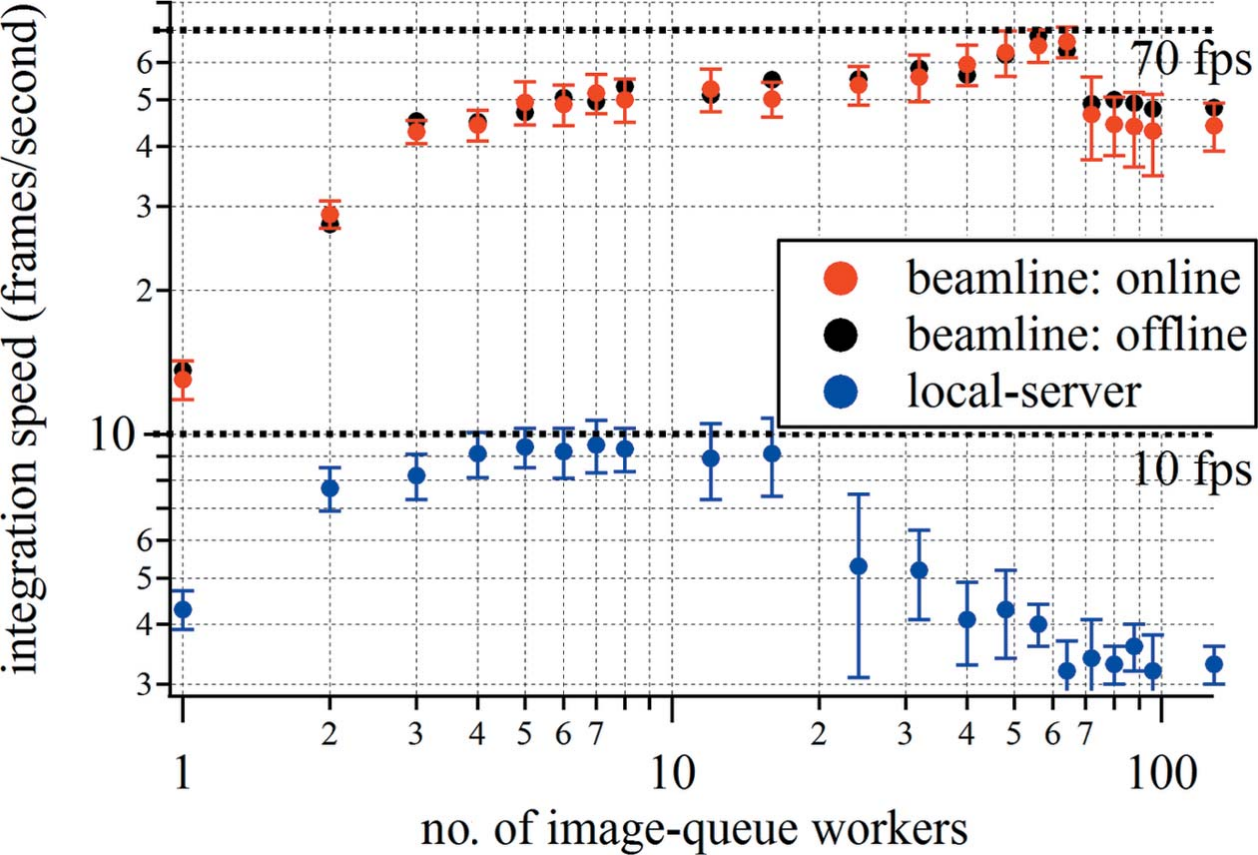
Results of the benchmarking tests of the *SAXSDOG* software on the Austrian SAXS beamline at the Elettra Sincrotrone storage ring. The integration speeds were determined from the time necessary to integrate 5000 images from a Pilatus 1M detector (4 MB per image). In ‘online’ mode, the image queue is filled by the FEEDER, and in ‘offline’ mode, the image queue is filled by the directory walker (see Fig. 3 ▸).

As seen in Fig. 8 ▸ (red markers – ‘online’), the best performance (66.3 ± 4.9 f.p.s.) was achieved when using 64 workers, although with only 8–16 workers integration speeds of approximately 50 f.p.s. were observed. The peak processing rate corresponds to a memory-access speed of ∼280 MB s^−1^, which is above the single-hard-drive hardware limit declared by the manufacturer of 250 MB s^−1^ (enabled by the RAID data-storage system, such that image files are unconsciously read from different hard drives). An increase of workers above 64 results in a drop in performance and is hence not recommended. The same performance measurements have been made in the ‘offline’ mode on the storage server, so when images have been acquired previously such that the image queue is not filled by the FEEDER but by the directory walker (see Fig. 3 ▸). These measurements (see black markers in Fig. 8 ▸) showed an appreciable performance difference compared with the ‘online’ integration mode. Data acquisition by the detector as well as transfer onto the beamline server prior to integration hence does not seem to affect the integration performance. A long-term test, processing approximately 300 000 images (corresponding to 1.2 TB of data), showed that integration speeds of approximately 60 f.p.s. (using 40 workers – corresponding to 240 MB s^−1^ memory-access speed) can be maintained for more than 1.3 h. When processing images on a normal workstation PC in ‘local-server’ mode (see subsection 3.4) the overall integration speed is significantly lower (see blue markers in Fig. 8 ▸): we achieve approximately 10 f.p.s. when using 4–16 image-queue workers. The memory-access rate of approximately 40 MB s^−1^ is half of the HDD’s hardware limit, suggesting that either CPU or RAM performance is limiting the overall integration speed.

Overall, the performance of the *SAXSDOG* pipeline implemented at the Austrian SAXS beamline of the Elettra storage ring is sufficient to process scattering images ‘online’, so within seconds after acquisition. The sustainable integration speed of approximately 60 f.p.s. (average processing time of 17 ms per image) ranks comparable to other software packages (Ashiotis *et al.*, 2015 ▸) for azimuthal integration, especially considering that this rate (i) can be maintained over hours, (ii) already includes data transfer from the detector to the long-term data-storage system and (iii) is just below the hardware limit of the hard drives (240 MB s^−1^ according to the manufacturer-specified limit of 250 MB s^−1^). For small data sets, for example, from laboratory machines, processing rates in the local-server mode are more than sufficient for azimuthal integration of SAXS data, making *SAXSDOG* an attractive choice also for not-beamline-related use.

## 6. Conclusion

In summary, we have designed, developed and implemented *SAXSDOG*: a software package for fast online integration of 2D scattering images. We show how the *SAXSDOG* suite is useful for two separate operation schemes: (1) the ‘local-server mode’ that can be run on standalone computers and (2) the ‘remote-server mode’ as used in the data pipeline of the Austrian SAXS beamline at the Elettra synchrotron. By optimizing the program for online (real-time) integration during *in situ* experiments, we reach peak integration performance at current hardware limits. Particular focus has been set on allowing operation via a graphical user interface, which sets all integration parameters, controls all ongoing server processes and shows the current integration progress, including image classifiers for preliminary data evaluation. The software (open-source code) can be used and is released free of charge under the GNU General Public License. We strongly encourage participation in further code development.

## Figures and Tables

**Table 1:** Mandatory parameters to be specified in the calibration

Name	Type	Unit	Description
geometry	Object	–	Includes information on the experimental geometry
- beamcenter	Array	Pixel	Position (vertical, horizontal) of the beam center on the detector
- detector distance	Number	mm	Sample-to-detector distance
- image size	Array	Pixel	Dimensions (vertical, horizontal) of the sensor
- pixel size	Array	µm	Pixel size (vertical, horizontal) on the detector
- tilt	Object	–	Includes information on the detector tilt
- - tilt rotation	Number	°	Angle of the tilt direction
- - tilt angle	Number	°	Angle between the primary beam and the normal of the detector
masks	Array	Object	List of masks to use for integration
- path to file	String	–	Path to mask file (supports *.msk files from *FIT2D*)
- oversampling	Number	Pixel	Oversampling/anti-aliasing factor for radial integration
- pix. p. rad. element	Number	Pixel	Width of each radial step in units of detector pixels
- q-start	Number	nm^−1^	Lower boundary for calculation of integral parameters
- q-stop	Number	nm^−1^	Upper boundary for calculation of integral parameters
wavelength	Number	Å	Wavelength of the X-ray beam
directory	Array	String	Directory to take into account for processing images
threads	Number	–	Number of parallel workers to use during image processing

**Table 2:** Parameters to be specified in the calibration to calculate the mean scattering intensity along a single slice

Name	Type	Unit	Description
slices	Array	Slice	Array of slice objects, as specified bellow
- direction	String	–	Direction of the slice on the detector plane (x or y)
- plane	String	–	Whether the slice direction is in-plane with the scattering surface or perpendicular to it (InPlane or Vertical)
- position	Number	Pixel	Pixel position at which to place the slice (*x* coordinate for *y* slice and *y* coordinate for *x* slice)
- margin	Number	Pixel	Number of pixels left and right from the position to include in the slice
- mask reference	Number	–	Pointer to the mask object (see Table 1 ▸) to use for integration
